# Seismic Model Parameter Optimization for Building Structures

**DOI:** 10.3390/s20071980

**Published:** 2020-04-01

**Authors:** Lengyel Károly, Ovidiu Stan, Liviu Miclea

**Affiliations:** Department of Automation, Faculty of Automation and Computer Science, Technical University of Cluj-Napoca, Memorandumului Str. 28, 400014 Cluj-Napoca, Romania; Karoly.Lengyel@student.utcluj.ro (L.K.); Liviu.Miclea@aut.utcluj.ro (L.M.)

**Keywords:** structural dynamic modeling, optimization, DE, PSO, parameter estimation, extended Kalman filter, inverted pendulum

## Abstract

Structural dynamic modeling is a key element in the analysis of building behavior for different environmental factors. Having this in mind, the authors propose a simple nonlinear model for studying the behavior of buildings in the case of earthquakes. Structural analysis is a key component of seismic design and evaluation. It began more than 100 years ago when seismic regulations adopted static analyzes with lateral loads of about 10% of the weight of the structure. Due to the dynamics and non-linear response of the structures, advanced analytical procedures were implemented over time. The authors’ approach is the following: having a nonlinear dynamic model (in this case, a multi-segment inverted pendulum on a cart with mass-spring-damper rotational joints) and at least two datasets of a building, the parameters of the building’s model are estimated using optimization algorithms: Particle Swarm Optimization (PSO) and Differential Evolution (DE). Not having much expertise on structural modeling, the present paper is focused on two aspects: the proposed model’s performance and the optimization algorithms performance. Results show that among these algorithms, the DE algorithm outperformed its counterpart in most situations. As for the model, the results show us that it performs well in prediction scenarios.

## 1. Introduction

Structural dynamic modeling of buildings has come a long way in the last 50 years, due to the competition of software development companies and the increased availability of computational resources. These technologies have evolved from simulating only prismatic beams to including geometrical and material nonlinearities [1]. From a control engineering perspective, these models have a particularly great importance when designing control systems for earthquake hazard mitigation. Having a good model exclusively for the above mentioned purpose can significantly improve the behavior of these systems [2].

The civil engineering field is imaginative, and it ranges from water-resources to structural design and analysis. Generally speaking, the problems in this field are unstructured and imprecise, influenced by a designer’s intuitions and past experiences. The conventional computing methods based on analytic or empirical relationships take time and are labor intensive when they are presented with real life problems. In addition, Soft Computing techniques (SC) based on the reasoning, intuition, conscience, and knowledge of an individual can be easily empowered to study, model, and analyze such problems [3,4].

Unlike conventional computing technology based on exact solutions, in SC, either independent or mutually complementary work supports engineering activities by utilizing the human mind’s cognitive behavior to achieve cost-effective solutions aimed at exploiting the trivial and uncertain nature of the problem in a given tolerance of imprecision to achieve a quick solution to a problem [5]. As a multidisciplinary field, SC employs a variety of complementary tools, including statistical, probability, and optimization tools.

According to Falcone et al., SC should be divided into two main domains [5]. The first one, approximate thinking, collects a set of knowledge-driven methods that sacrifice health or completeness in order to achieve a substantial speed of thinking. The second one, randomized search, is also a family of digitally optimized techniques, such as direct search, free derivative search, or black-box search, which work by moving iteratively to better positions in the search space, which are sampled from a surrounding hyper sphere.

Earthquake engineering can be described as the civil engineering branch devoted to reducing the risks of an earthquake. An earthquake is the moment when the Earth’s surface is shaking, which is caused by moving interactions on the boundary of a plate [6]. The sudden release of energy, called seismic waves, kills thousands of people and destroys many buildings. In this narrow context, earthquake engineering examines problems that occur when the earthquake occurs and seeks methods that minimize the damage caused by its activities. The first leads to the prediction of an earthquake, while the second leads to the optimal design of objects’ seismic performance. A whole range of earthquake engineering problems have arisen that are suitable to solve by SC [7,8]. The focus of SC in earthquake engineering is on solving two types of problems: the search for the best seismic structural design (system analysis); data analysis for earthquake prediction (modeling and simulation). In order to achieve earthquake safety, seismic design optimization addresses passive and active structures [9].

The goal of this project is to measure the performances of different variants of DE and PSO in optimizing the parameters in a proposed seismic model for building structures that is lightweight enough to be used for different applications that require easy computation and reliability. The authors will analyze the convergence speed and other indicators of the algorithms’ performance and will compare the two algorithms in this use case. The achieved model will also be tested in two scenarios: simulation and prediction. The prediction will be performed using an extended Kalman filter.

**Figure 1:**
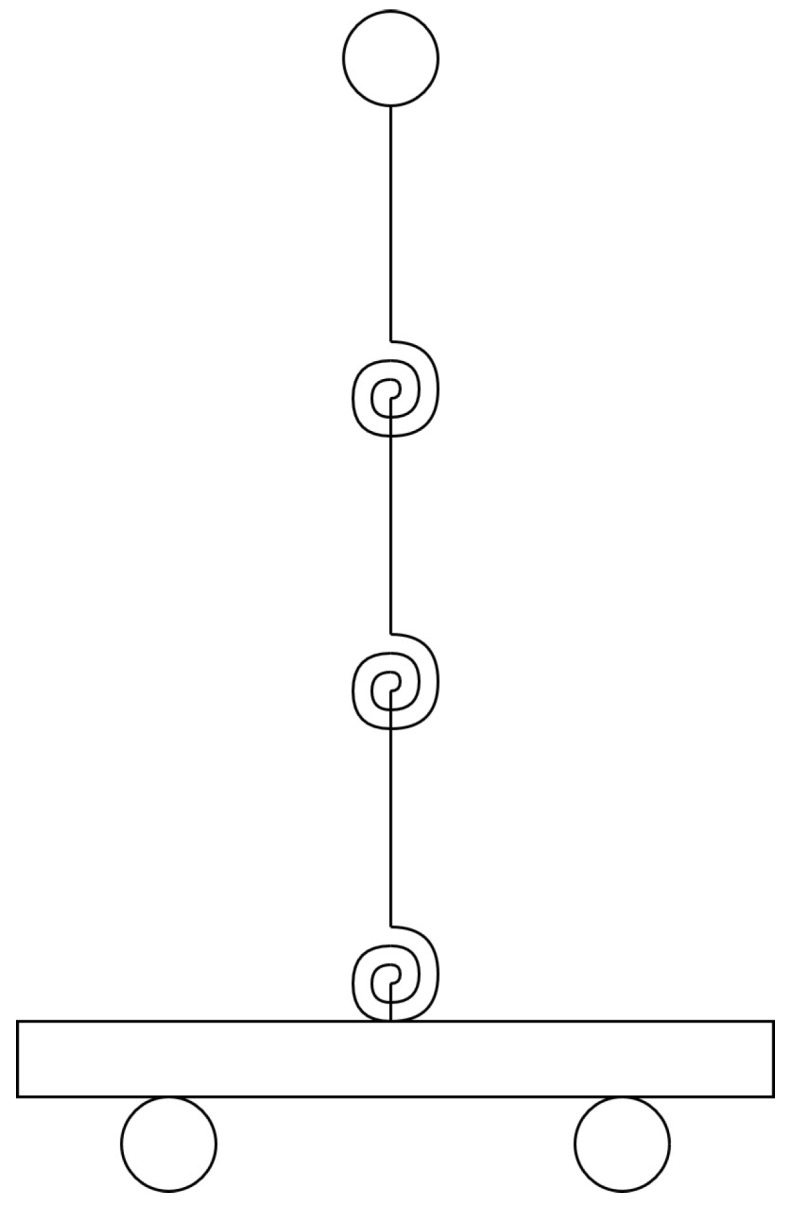
Three segment inverted pendulum on a cart with mass-spring-damper joints.

The proposed model is a multiple segment inverted pendulum on a cart with mass-spring-damper rotational joints, as illustrated in Figure 1.

The bibliographic search for this project can be divided into the following sections, regarding the field in which it was performed:

- System identification and parameter estimation
- Kinetic modeling
- Kalman filter
- Optimization

### 1.1. Context

Most buildings are deformed significantly when strong earthquakes affect them. One of the factors contributing to quantitative thinking beyond the elastic response of structures is the gap between measured ground speed and the seismic design forces defined in codes [10]. There is however a long way to go prior to more advanced seismic codes for the explicit nonlinear analysis. The use of force reducing factors was initially the most popular approach, and today, this approach is still popular. Although this concept of taking inelastic behavior into account has been useful for many decades in linear analysis, it is only possible to give a realistic assessment of structural behavior in the inelastic range through non-linear analysis. A gradual implementation of nonlinear analyses, which should be explicitly able to simulate the second fundamental feature of a structured answer to strong seismic movement of the Earth, namely the inelastic behavior, characterizes present developments of the analysis processes in seismic codes. Data on the structure need to be known for such nonlinear analyses, which makes them well suited for analyzing existing structures. For newly designed structures, a preliminary design must be carried out before a nonlinear analysis is started [11].

### 1.2. System Identification and Parameter Estimation

Due to the uncertainty, time-lagging, multi-variable couplings, and the limitations between the input and output, traditional model control methods are becoming increasingly difficult to control complex processes correctly in the rapid development of modern industry. Due to the complex structure, different parameters and time variations for industrial applications, this is a challenge for traditional identification methods, particularly in multivariate systems. Methods for identifying multi-variable systems date back to the 1960s, but the majority of methods for identifying them require noise-free observations. Together with their high calculation costs, this makes them difficult to apply in practice [12]. In view of the above problems, many scientists proposed that a polynomial matrix be substituted for the state space model, to define the multi-variable system [13].

Some researchers then proposed the Hankel matrix-based methods for row subspace identification. The first step in this method is to obtain the system’s increased observability matrix (or status sequence) and then calculate the parameter matrix of each sub-space. Multi-variable output error status [13], sub-space state-space identification numerical algorithms [14], and canonical variate analyses [15] are the main representative techniques.

Input signal selection is an important factor in system identification, as stated in [16], where the authors discussed the importance of the input signal selection and explained, briefly, a few types of input signals for system identification. The discussed signals were: the step, pseudo random binary sequence, auto-regressive moving average process, and sum of sinusoids. Based on this information and from prior knowledge, our choices for identifying signals were the step and PRBS signals. In the same book, in Chapter 1, the authors discussed the influence of data feedback on the identification performances. This is of great importance, because our system has strong feedback. They concluded that by having feedback in a system, it can make it unidentifiable. However, by having a reference signal, the previously mentioned problem disappears, affecting the identification performance.

Extensive research has also been done on PSO’s performance compared to that of GA. One example is [17], where the authors discussed the performance of the PSO algorithm compared to that of Genetic Algorithms (GA) in system identification. Their case was a nonlinear model, and the experiment was performed online. They concluded that this type of algorithm is an efficient tool in nonlinear system identification, producing similar and better results than GA, having the advantage of low computational cost and faster convergence. Worden et al. also recently arrived at the same conclusion about nonlinear system identification [18]. The identification of non-linear systems involves much more than linear identification. The following aspects contribute to this observation: non-linear models live in a multiplex system of a greater size, while linear models live in easier to characterize simple hyperplanes; in non-linear system identification, structural model errors are frequently inevitable, and this affects the three main choices: experiment design, model selection, and cost-function selection; entering noise before non-linearity requires new numerical tools to solve the problem of optimization [19]. Moreover, extensive research has been done to compare parameter estimation capabilities to PSO variations like PSO, APSO, and Quantum behaved PSO (QPSO) [20,21]. The nonlinear model types on which the experiments are performed are the Hammerstein and Wiener models. Their conclusion was that using swarm intelligence, such as modifying the original algorithm, improved the parameter estimation performance. Other variations of the PSO algorithm have been studied for system identification; for example, the PSO-QI algorithm was discussed in [22], where the authors of the paper analyzed the use of the PSO-QI algorithm for system identification, which was compared to to the classic PSO and DE. They concluded that for system identification, the modified algorithm was the best among the three because of its fast convergence.

Research has also been done when using DE for system identification and parameter estimation in systems. The work in [23] discussed the optimal approximation of linear systems using a Differential Evolution (DE) algorithm. The authors incorporated a search space expansion scheme in order to overcome the difficulty of specifying proper intervals for initializing the DE search. Besides PSO, DE variations have also been studied for these tasks, for example in [24], where the authors discussed a hybrid DE algorithm for nonlinear parameter estimation of kinetic systems. In this article, the authors combined the DE algorithm with the Gauss–Newton method. Basically, the DE was used to provide a good initial point for the Gauss–Newton algorithm, which then found the absolute minimum. Their conclusion was that this approach was an effective one for this kind of task.

### 1.3. Kinematic and Kinetic Modeling

Kinematics refers to the study of object movement without taking into account the forces acting on it. An *n* segmented inverted pendulum can be considered as a kinematic chain (parts serially connected by joints). Each element can be defined as a rigid body defining a geometric relationship between two joints [25]. Based on these assumptions and on Natsakis’ course [26], an n segmented inverted pendulum kinematic model can be looked at as a one degree of freedom joint series of n elements connected on n−1 links with the length considered to be zero. The axis of a joint is determined by the rotation of link *i* in relation to link i−1. The distance between two different axes can be measured by determining the common perpendicular on them. If two axes are parallel, they can describe an infinite number of common perpendiculars, but all with the same length.

The forward kinematics model describes the relation between variable orientation or displacement inputs for each joint and the position and orientation of the end effector, represented in a 4 × 4 homogeneous transformation matrix. There are several approaches for computing the forward kinematics model, but in this paper, we will discuss the Denavit–Hartenberg convention [27,28]. The coordinate system for each link is defined by the following rules: the rotation axis of the joint represents the Z axis; the perpendicular on the plane formed by the current joint Z axis and the following joint Z axis represents the current joint X axis. The convention is based on four parameters, set out in Table 1 after defining the coordinate system.

Olav et al. proposed the Lagrangian approach to determine the kinetic model [29], but the advantages [30] and limitations of this type of model [31] can be easily found in the literature.

### 1.4. Kalman Filter

In 1960, R. E. Kalman introduced his famous discrete data filtering technique [32]. The Kalman filter is basically a set of mathematical equations that provides an efficient way of computing the least squares problem using a recursive method. It is very powerful, because it can estimate the future, the present, and the past states of a system, even if its true nature is unknown. The original algorithm is suitable for linear state space models. For nonlinear state space models, the extended Kalman filter was introduced. This algorithm linearizes the operation around a current estimate with the help of partial derivatives [33].

The Kalman filter, even though it was introduced in 1960, is widely used and lately has provided one of the most common ways to minimize the disadvantages [34] associated with strap-down inertial navigation systems [35]. The filtering method requires an accurate dynamic model [36] and observed integration model, including an inertial sensor error stochastic model and a priori details on content regression coefficients between the two systems [37]. However, there are several inconsistencies, as follows: the difference in the linearization approach; precise stochastic modeling that cannot accurately model sensors; the need for stochastic parameters to be adjusted. Each needs a new a priori sensor system and information [38]. In addition, some filtering methods [39,40] were successfully applied.

In the fine alignment process, the error of the inertial sensors is estimated and compensated using the optimum estimation algorithm in order to improve the accuracy of the initial attitude matrix. The most frequently used estimates are based on a Kalman filter, which can handle only linear systems and requires accurate information about noise statistics [41].

Petritoli et al. concentrated on the well-known data fission with integrity monitoring, low cost sensors, and a low energy consumption computer; however, they did not take into account the aging effects of such low cost sensors in depth [42].

The Kalman filter is relatively less mathematically complicated and easier to deploy compared to the other filters, such as the particle filter. However, the capacity of the Kalman filter to position nonlinear integrated systems accurately is limited [42,43]. However, for example, there are also some advantages of using this approach [44]. Eom et al. established a method for the improvement of physical estimates using multiphysical models and Kalman data fusion filters by processing raw measurements within a sensor [45].

### 1.5. Optimization

Optimization is the most appropriate solution, with a set of restrictions. It is in the nature of humans to try to find an optimal solution for each problem. Due to advances in computing technology and algorithms, large optimization problems can be very easily solved. However, there still exist a great number of problems whose search is very broad, and the classical optimization techniques cannot trace these problems. Metaheuristics are highly useful and always provide an optimum solution to solve these difficult optimization problems [46].

#### 1.5.1. Particle Swarm Optimization

Particle Swarm Optimization (PSO) is one of the optimization methods used in this project and was first presented in [47] by Kennedy and Eberhart. PSO is a swarm intelligence evolutionary algorithm that simulates bird and fish predatory behavior, and due to PSOs being simple in structure, strong maneuverability, easy implementation and other characteristics, they have attracted much attention from scientists and researchers. proposed a new optimization algorithm called particle swarm optimization, which according to the authors, lied somewhere between genetic algorithms and evolutionary algorithms. They proposed a very simple, but effective algorithm that could optimize a wide variety of functions. The developments, applications, and resources of the PSO algorithm, based on a computer science and engineering perspective, were described in [48]. This work also briefly described the inertia weight parameter and the possible need for a constriction factor. PSO has so far been applied successfully in many areas [49,50,51,52], and some improved PSO versions have also been studied [52,53,54,55,56]. Basically, the PSO algorithm has been used to find an optimum search space in complex areas by interacting with people in a particle population [57]. A number of problems such as artificial neural network training [58], fuzzy logic control [59], or pattern classification [60] have been successfully addressed.

In [61], a modification of the PSO algorithm was proposed, called adaptive particle swarm optimization, which will be implemented in this project. Their modified algorithm enables automatic control of certain algorithm parameters such as inertia weights and acceleration constants. Other modifications of the original algorithm have been discussed. For example, the work in [62] discussed a modification of the PSO algorithm called the quantum behaved PSO, which relies on the QPSO, but in addition, uses a recombination operator based on interpolation, which generates a new vector of possible solutions. In this article, the author also briefly described the classical QPSO algorithm, which will be implemented in this project with the proposed modifications.

Parameter selection has also been subject to extensive research. In [63], the author discussed different parameter selection methods for the PSO algorithm, including previous proposals from researchers, while the work in [64] also discussed the best PSO parameters for different situations.

Shi and Eberhart [65] were the ones that first introduced the basic evolution equations of the algorithm with the inertial weight as the relatively important PSO control parameter, and some research has since been undertaken on the inertia weight’s influence on optimization performance. In accordance with Bayesian theory, Zhang et al. designed an adaptive adjustment strategy for the weight of inertia [66], while at the same time fully applying its historical position. Although the convergence accuracy of this enhanced PSO was greater, the rate of convergence was slow.

#### 1.5.2. Differential Evolution

In [67], the authors proposed a new global optimization method called differential evolution. They concluded that this algorithm was superior to Adaptive Simulated Annealing (ASA), as well as the Annealed Nelder–Mead approach (ANM). It was also superior in terms of the ease of use, since only two parameters had to be chosen from a well-defined interval. DE also has its variations, one being the EDE, which is different in terms of the trial population generation.

DE is a search algorithm based on a population that works with a collection of solutions modified over the generations to find better solutions through selection, generation, and replacement schemes [46,68]. DE is an evolutionary approach to complex problems with optimization. The DE is a simple and popular stochastic algorithm based on the population. When measured against the benchmark problem and actual performance optimization issues, DE outperformed other competitive evolutionary algorithms. DE’s main drawback, like other stochastic optimization algorithms, is early convergence and stagnation at suboptimal points. Unlike many other evolutionary computation techniques, basic DE is a very simple algorithm, whose implementation in any standard programming language requires just a couple of lines of code. However, while optimizing a wide range of objective functions, DE shows remarkable performance in terms of ultimate precision, computational speed, and robustness [69].

## 2. Materials and Methods

### 2.1. Quanser Shake Table II

The Quanser Shake Table II (STII) is a shake table device for training, which had originally been developed by the University Consortium on Instructional Shake Tables (UCIST). It can be used for educational [70] and research purposes in various topics such as mechanical, aerospace [71] and civil engineering structural dynamics [72], vibration isolation [73], and feedback control [74].

**Figure 2:**
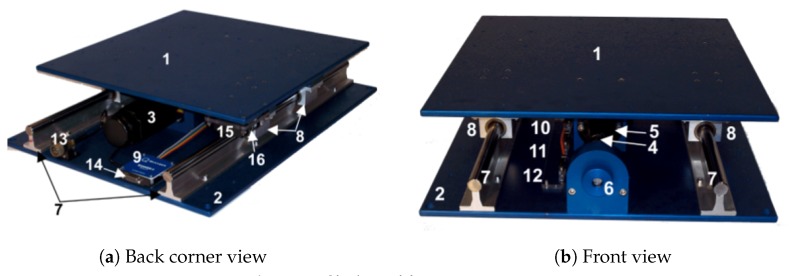
Shake Table II components.

The STII (as shown in Figure 2) [75] has a maximum load of 7.5 kg at an acceleration of 2.5 g (24.525 m/s${}^{2}$). The staßge is dispatched on two metal shafts with a hard ground with linear rows that allow smooth linear movements with low path deflection. From the center, the stage is able to move ±7.62 cm or ±3 inches (total journey of 15.24 cm). A robust ball screw is connected to a 400 Watt three phase brushless DC actuator. The electric motor has a built-in high-resolution encoder for measuring the stage position at a resolution of 3.10 μm. To measure the step acceleration directly, an analog accelerometer is mounted directly on the stage. Figure 2 lays out the components given in Table 2. Figure 2a provides a cornered perspective of the back, while Figure 2b provides a front view of the shake table.

### 2.2. Initial Data

**Figure 3:**
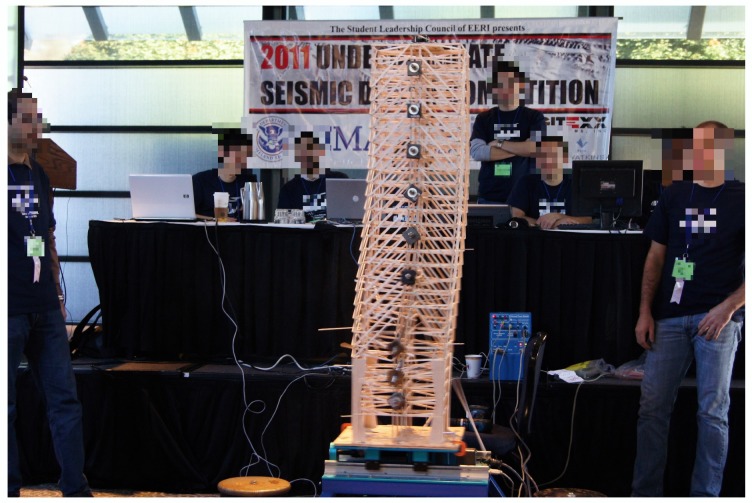
Shake Table II in action.

The experiments were performed on a Quanser Shake Table II. The balsa structure was tested on the above mentioned shake table for a given earthquake, and the logged data were the table acceleration reference, the actual acceleration of the table, and the accelerations of the top of the structure. An illustration of the balsa structure, while being tested, can be seen in Figure 3.

**Figure 4:**
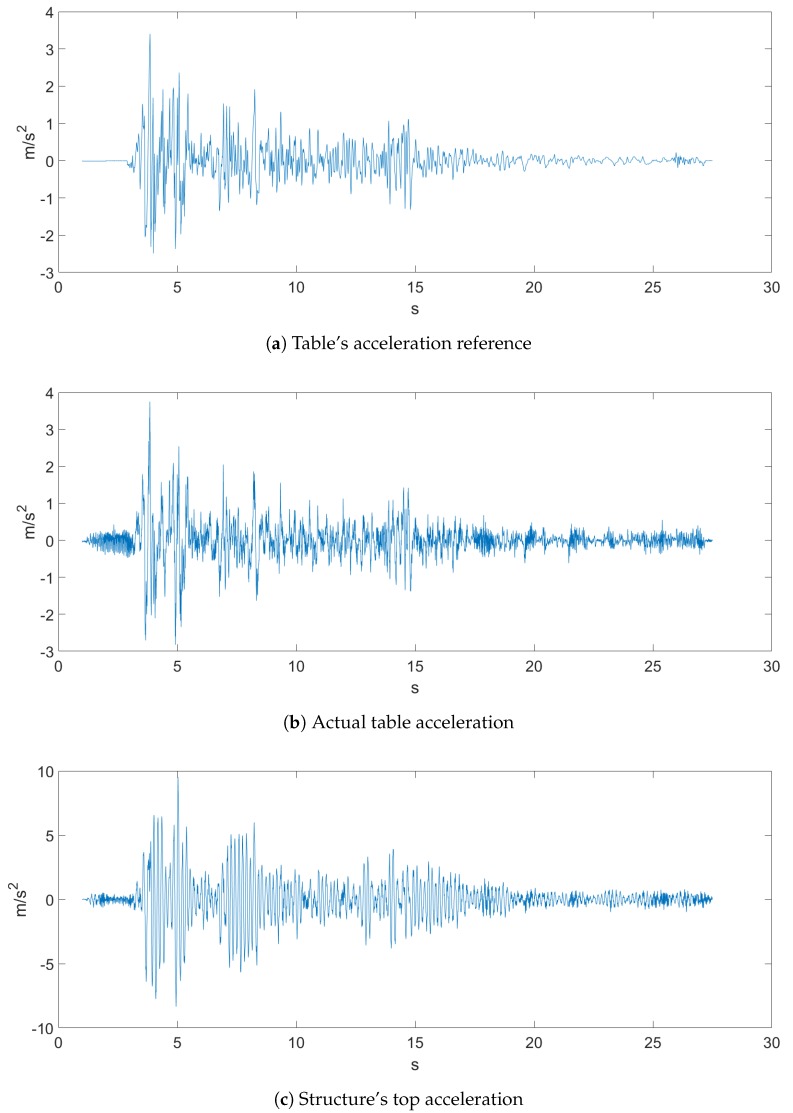
Ground Motion 1.

**Figure 5:**
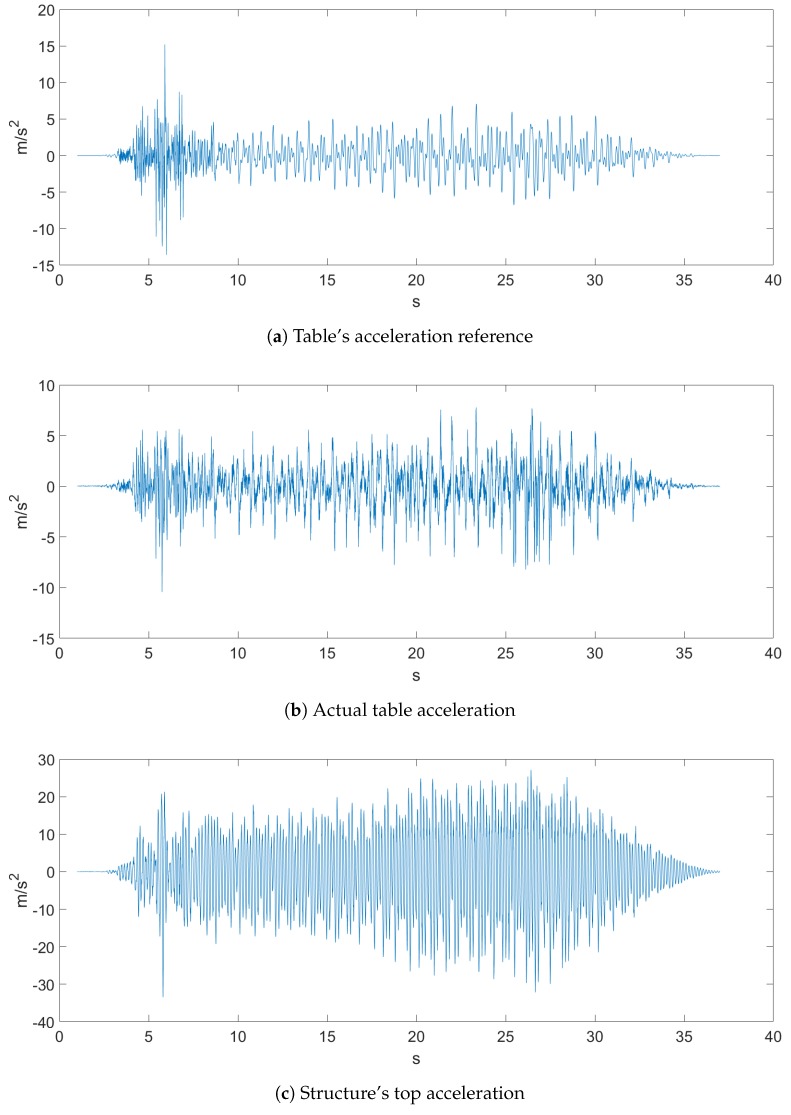
Ground Motion 2.

The sampled data came with a sampling rate of 500 Hz, and both earthquakes, which can be seen in Figure 4 and Figure 5, were the ones proposed in the Seismic Design Competition 2019 organized by the EERI Student Leadership Council https://slc.eeri.org/2019-seismic-design-competition/.

Figure 4 represents Ground Motion 1 of a recorded earthquake. Figure 4a depicts the table acceleration reference, which was the desired displacement of the table, while Figure 4b is the actual table acceleration that was measured with an accelerometer. Figure 5 depicts Ground Motion 2, which is a different, more aggressive earthquake.

The measurements were collected using the Quanser Shake Table II equipment, including both the hardware and software part. The parameters were set to the ones recommended in [75]. The shake table controller also needed the velocity and position setpoints, so for our experiments, we generated data structures from the raw data, which contained:

- Sampling time
- Top accelerations
- Actual table’s acceleration
- Table’s acceleration reference
- Table’s velocity reference
- Table’s position reference

### 2.3. Shake Table Controller

The shake table controller design was described in [75]. According to pages 13–15, the controller consisted of a proportional derivative and feed forward controller.

#### 2.3.1. Table Model

The actual table’s transfer function can be written in the following format:

(1) $$H\left(s\right)=\frac{X(s)}{I(s)}=\frac{1}{K_{f}s^{2}}$$

where X(s) is the table displacement, I(s) is the motor current, and:

(2) $$Kf=\frac{M_{t}P_{b}}{K_{t}}$$

is the model gain, where $M_{t}$ is the total mass being moved by the motor, $P_{b}$ is the pitch of the ball screw, and $K_{t}$ is the current-torque coefficient [75].

Since our Lagrangian model, which is discussed later, needed force at the input, the model transfer function becomes:

(3) $$H\left(s\right)=\frac{X(s)}{F(s)}=\frac{1}{M_{t}s^{2}}$$

#### 2.3.2. Table Controller

As described in [75], the proportional derivative plus feed forward controller had the following form:

(4) $$I\left(s\right)=K_{p}e\left(s\right)+K_{d}e\left(s\right)s+K_{f}e\left(s\right)s^{2}$$

where $K_{p}$, $K_{d}$, and $K_{f}$ are the PD+FF controller gains. Considering that the feed forward element was zero and substituting Equation (1), in order to find the closed-loop transfer function, the equation becomes:

(5) $$K_{f}s^{2}X\left(s\right)=K_{p}\left(X_{d}\left(s\right)-X\left(s\right)\right)+K_{d}s\left(b_{sd}X_{d}\left(s\right)-X\left(s\right)\right)$$

where $b_{sd}$ is the velocity weight coefficient and $X_{d}$ is the table position reference, which yields the closed-loop transfer function:

(6) $$H\left(s\right)=\frac{X(s)}{X_{d}\left(s\right)}=\frac{K_{p}+K_{d}b_{sd}s}{K_{f}\left(s^{2}+\frac{K_{d}}{K_{f}}s+\frac{K_{p}}{K_{f}}\right)}$$

which is equivalent to a standard second order system if $b_{sd}=0$:

(7) $$H\left(s\right)=\frac{\omega^{2}}{s^{2}+2\zeta \omega s+\omega^{2}}$$

The shake table used in our experiments was calibrated to have a natural frequency of ω=15·2·π and a damping factor of ζ=0.75. To match this, we needed the following control gains: $K_{p}=K_{f}\omega^{2}$ and $K_{d}=2\zeta \omega K_{f}$. Since $K_{f}=0.5492$, we obtained $K_{d}=77.6344$ and $K_{p}=4.8779$.

To match the closed-loop system described above for our system, the controller is written in the following form:

(8) $$F\left(s\right)=K_{p}e\left(s\right)+K_{d}e\left(s\right)s+K_{f}e\left(s\right)s^{2}$$

where substituting Equation (3) and considering the feed forward element to be zero, it becomes:

(9) $$M_{t}s^{2}X\left(s\right)=K_{p}\left(X_{d}\left(s\right)-X\left(s\right)\right)+K_{d}s\left(b_{sd}X_{d}\left(s\right)-X\left(s\right)\right)$$

yielding the closed-loop transfer function:

(10) $$H\left(s\right)=\frac{X(s)}{X_{d}\left(s\right)}=\frac{K_{p}+K_{d}b_{sd}s}{M_{t}\left(s^{2}+\frac{K_{d}}{M_{t}}s+\frac{K_{p}}{M_{t}}\right)}$$

Considering the velocity weight coefficient to be zero, the transfer function matches the standard second order system described in Equation (7). In order to match the same natural frequency and damping factor as stated above, the following control gains were necessary: $K_{p}=M_{t}\omega^{2}$ and $K_{d}=2\zeta \omega M_{t}$. Since $K_{f}=M_{t}=7.74$ (the mass of the table), we obtained $K_{d}=1094.21$ and $K_{p}=$ 68,751.66.

#### 2.3.3. Filters

In the shake table laboratory guide [75], it was also stated the that direct derivatives from the encoder were not taken in order to avoid noisy signals; instead, the table displacement was filtered with the following second order filters to obtain the stage’s velocity and acceleration:

(11) $$H_{f1}\left(s\right)=\frac{{\dot{X}}_{f}\left(s\right)}{X(s)}=\frac{\omega_{d}^{2}s}{s^{2}+2\zeta_{d}\omega_{d}s+\omega_{d}^{2}}$$

(12) $$H_{f2}\left(s\right)=\frac{{\ddot{X}}_{f}\left(s\right)}{X(s)}=\frac{\omega_{f}^{2}s^{2}}{s^{2}+2\zeta_{f}\omega_{f}s+\omega_{f}^{2}}$$

where $\omega_{d}=2\cdot \pi \cdot 50$, $\zeta_{d}=0.9$, $\omega_{f}=2\cdot \pi \cdot 25$, $\zeta_{f}=0.9$, ${\dot{X}}_{f}\left(s\right)$ is the filtered velocity and ${\ddot{X}}_{f}\left(s\right)$ is the filtered acceleration.

#### 2.3.4. Discretization

The controller Equation (8) can also be written in the following form:

(13) $$F\left(s\right)=K_{p}\left(X_{d}\left(s\right)-X\left(s\right)\right)+K_{d}\left({\dot{X}}_{d}-X\left(s\right)H_{f1}\left(s\right)\right)+K_{f}\left({\ddot{X}}_{d}\left(s\right)-X\left(s\right)H_{f2}\left(s\right)\right)$$

that is:

(14) $$F\left(s\right)=K_{p}\left(X_{d}\left(s\right)-X\left(s\right)\right)+K_{d}\left({\dot{X}}_{d}\left(s\right)-{\dot{X}}_{f}\left(s\right)\right)+K_{f}\left({\ddot{X}}_{d}\left(s\right)-{\ddot{X}}_{f}\left(s\right)\right)$$

and by applying the *z*-transformation:

(15) $$F\left(z\right)=K_{p}\left(X_{d}\left(z\right)-X\left(z\right)\right)+K_{d}\left({\dot{X}}_{d}\left(z\right)-{\dot{X}}_{f}\left(z\right)\right)+K_{f}\left({\ddot{X}}_{d}\left(z\right)-{\ddot{X}}_{f}\left(z\right)\right)$$

Furthermore, by applying the *z*-transform to Equations (11) and (12) with the zero order hold method and a sampling interval of 0.002 s, we obtain:

(16) $$H_{f1}\left(z\right)=\frac{110.7z-110.7}{z^{2}-1.094z+0.3227}$$

(17) $$H_{f2}\left(z\right)=\frac{2.467\cdot 10^{4}z^{2}-4.834\cdot 10^{4}z+2.366\cdot 10^{4}}{z^{2}-1.889z+0.8931}$$

which are equivalent to:

(18) $${\dot{X}}_{f}\left(z\right)=1.094{\dot{X}}_{f}\left(z\right)z^{-1}-0.3227{\dot{X}}_{f}\left(z\right)z^{-2}+110.7X\left(z\right)z^{-1}-110.7X\left(z\right)z^{-2}$$

(19) $$\begin{array}{c} {\ddot{X}}_{f}\left(z\right)=1.889{\ddot{X}}_{f}\left(z\right)z^{-1}-0.8931{\ddot{X}}_{f}\left(z\right)z^{-2}+2.467\cdot 10^{4}X\left(z\right)\\ -4.834\cdot 10^{4}X\left(z\right)z^{-1}+2.366\cdot 10^{4}X\left(z\right)z^{-2} \end{array}$$

These yield the final controller’s equation:

(20) $$\begin{array}{c} F\left(k\right)=K_{p}\left(X_{d}\left(k\right)-X\left(k\right)\right)+K_{d}\left({\dot{X}}_{d}\left(k\right)-{\dot{X}}_{f}\left(k\right)\right)+K_{f}\left({\ddot{X}}_{d}\left(k\right)-{\ddot{X}}_{f}\left(k\right)\right),\\ {\dot{X}}_{f}\left(k\right)=1.094{\dot{X}}_{f}\left(k-1\right)-0.3227{\dot{X}}_{f}\left(k-2\right)+110.7X\left(k-1\right)-110.7X\left(k-2\right)\\ {\ddot{X}}_{f}\left(z\right)=1.889{\ddot{X}}_{f}\left(k-1\right)-0.8931{\ddot{X}}_{f}\left(k-2\right)+2.467\cdot 10^{4}X\left(k\right)-\\ 4.834\cdot 10^{4}X\left(k-1\right)+2.366\cdot 10^{4}X\left(k-2\right) \end{array}$$

**Figure 6:**
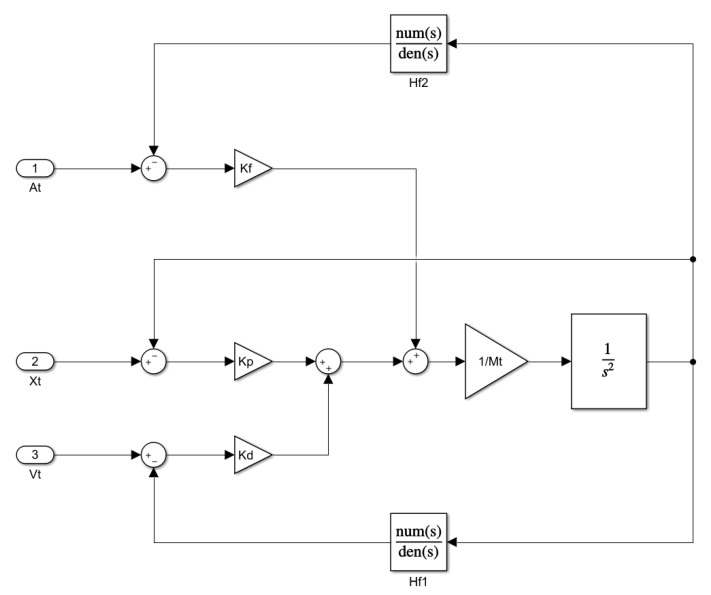
Controller’s block diagram.

A block diagram of the shake table controller can be seen in Figure 6.

### 2.4. Forward Kinematics

#### Denavit–Hartenberg Parameters

**Figure 7:**
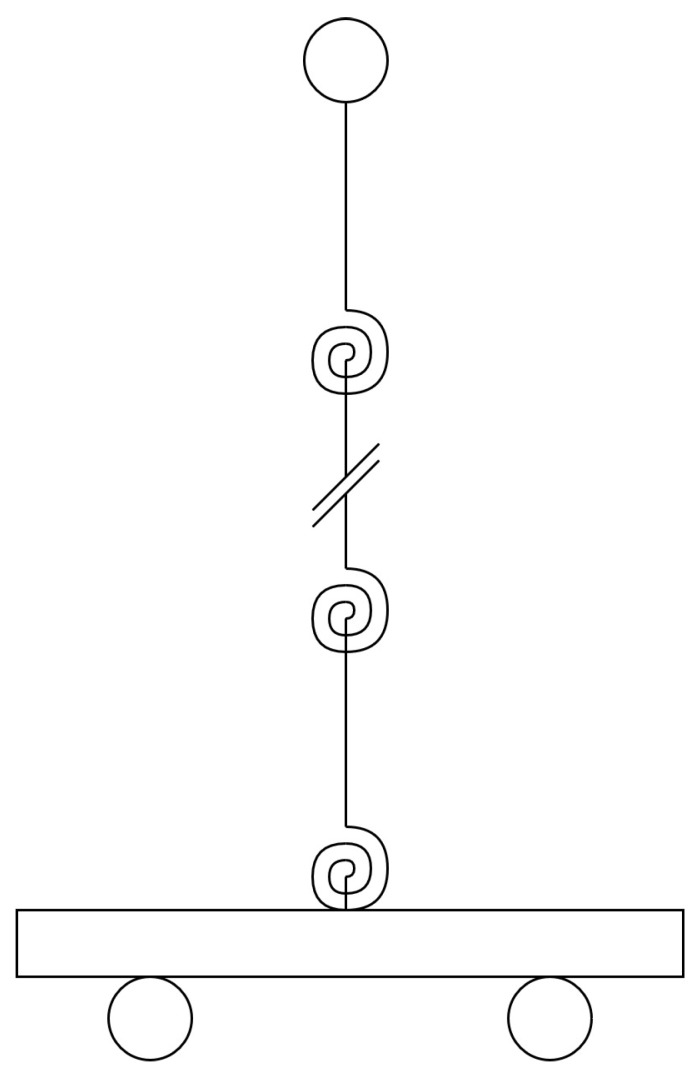
N segment inverted pendulum on a cart with mass-spring-damper joints.

For a multi-segment inverted pendulum on a cart, as seen in Figure 7, the Denavit-Hartenberg (DH) parameters can be seen in Table 3, where $q_{i}$i=0,1,…,n are the joints and $l_{i}$i=0,1,…,n are the segment lengths.

### 2.5. Forward Kinematic Model

According to [26], having the DH parameters, the direct geometric model [26] is:

(21) $$R_{i}^{i+1}=Rot\left(x,\alpha_{i}\right)\cdot Trans\left(x,r_{i}\right)\cdot Rot\left(z,\theta_{i}\right)\cdot Trans\left(z,d_{i}\right)$$

that is:

(22) $$R_{i}^{i+1}=\left[\begin{array}{cccc} \cos\theta_{i} & -\sin\theta_{i} & 0 & r_{i}\\ \sin\theta_{i}\cos\alpha_{i} & \cos\theta_{i}\cos\alpha_{i} & -\sin\alpha_{i} & -d_{i}\sin\alpha_{i}\\ \sin\theta_{i}\sin\alpha_{i} & \cos\theta_{i}\sin\alpha_{i} & \cos\alpha_{i} & d_{i}\cos\alpha_{i}\\ 0 & 0 & 0 & 1 \end{array}\right]$$

In our case, it simplifies to:

(23) $$\begin{array}{c} R_{0}^{1}=\left[\begin{array}{cccc} 0 & 1 & 0 & 0\\ 0 & 0 & 1 & q_{0}\\ 1 & 0 & 0 & 0\\ 0 & 0 & 0 & 1 \end{array}\right]R_{1}^{2}=\left[\begin{array}{cccc} \cos q_{1} & -\sin q_{1} & 0 & 0\\ 0 & 0 & -1 & 0\\ \sin q_{1} & \cos q_{1} & 0 & 0\\ 0 & 0 & 0 & 1 \end{array}\right]\\ R_{i}^{i+1}=\left[\begin{array}{cccc} \cos q_{i} & -\sin q_{i} & 0 & l_{i-1}\\ \sin q_{i} & \cos q_{i} & 0 & 0\\ 0 & 0 & 1 & 0\\ 0 & 0 & 0 & 1 \end{array}\right] \end{array}$$

for i=2,3,…,n.

#### Inverse Kinematics Model and the Jacobian

The inverse kinematics model and the Jacobian will not be calculated analytically, because this is too complex and not the purpose of this project; however, we will mention them in the upcoming sections. A brief description [26] of the inverse kinematics model is Equation (24), whereas Equation (25) is that for the Jacobian.

(24) $$\begin{array}{c} q=R_{i}^{0}\left(P_{x},P_{y},P_{z}\right)\\ q={\left[q_{1},q_{2},\ldots ,q_{i}\right]}^{T}\\ i=1,2,\ldots ,n \end{array}$$

(25) $$\begin{array}{c} \xi =J\dot{q}\\ \xi ={\left[\dot{x},\dot{y},\dot{z},\dot{\omega_{x}},\dot{\omega_{y}},\dot{\omega_{z}}\right]}^{T} \end{array}$$

where ξ denotes the linear and angular velocities of the end-effector, in our case the top of our model, and *q* denotes the joint positions.

### 2.6. Dynamic Modeling

The dynamic model of the kinematic chain described above was obtained using a Lagrangian-based approach, because it is suitable for complex kinetic chains. This type of dynamic model is based on substituting the Lagrangian of the system, Equation (26), into Equation (27):

(26) L=K−P

(27) $$\frac{d}{dt}\frac{\partial L}{\partial \dot{q}}-\frac{\partial L}{\partial q}=\tau$$

Equation (27) can also be written in a more condensed form [26]:

(28) $$\begin{array}{c} D\left(q\right)\ddot{q}+C\left(q,\dot{q}\right)\dot{q}+G\left(q\right)=\tau \\ D\left(q\right)=\sum_{i=1}^{n}\left[m_{i}J_{vi}^{T}J_{vi}+J_{\omega i}^{T}R_{i}I_{i}R_{i}^{T}J_{\omega i}\right]\\ C_{kj}\left(q\right)=\sum_{i=1}^{n}\frac{1}{2}\left(\frac{\partial d_{kj}}{\partial q_{j}}+\frac{\partial d_{ki}}{\partial q_{j}}-\frac{\partial d_{ij}}{\partial q_{k}}\right)\\ G(q)=\frac{\partial P}{\partial q}\\ P=\sum_{i=1}^{n}gh_{i}m_{i} \end{array}$$

where $I_{i}$ is the inertia tensor matrix of a joint and $h_{i}$ is the height of the joint related mass. The *D* matrix contains the terms related to the inertia of the system; *C* contains the terms related to the centrifugal and Coriolis terms; and the *G* matrix contains the terms related to the potential energies of the system; in our case, gravity. However, our system contained elastic and viscous damping forces due to the mass-spring-damper joint, which is why we introduced the *K* and *B* matrices, so that Equation (28) becomes:

(29) $$\begin{array}{c} D\left(q\right)\ddot{q}+C\left(q,\dot{q}\right)\dot{q}+G\left(q\right)+Kq+B\dot{q}=\tau \\ K=\left[\begin{array}{cccc} 0 & 0 & \ldots & 0\\ 0 & k_{1} & \ldots & 0\\ . & . & \ldots & .\\ . & . & \ldots & .\\ . & . & \ldots & .\\ 0 & 0 & \ldots & k_{n} \end{array}\right]B=\left[\begin{array}{cccc} 0 & 0 & \ldots & 0\\ 0 & b_{1} & \ldots & 0\\ . & . & \ldots & .\\ . & . & \ldots & .\\ . & . & \ldots & .\\ 0 & 0 & \ldots & b_{n} \end{array}\right] \end{array}$$

where $k_{i}$ are the elastic coefficients and $b_{i}$ are the viscous damping coefficients, i=1,…,n. This can be written in a more usable format:

(30) $$\ddot{q}=D^{-1}\left(q\right)\left(\tau -C\left(q,\dot{q}\right)\dot{q}-G\left(q\right)-Kq-B\dot{q}\right)$$

In our project matrices, *D*, *C*, and *G* not only depended on *q* and $\dot{q}$, but also on *l*, the segment lengths, and *m*, the segment’s weights, because these are also parameters that have to be estimated later.

#### 2.6.1. Continuous Model

Equation (30) can also be written in a state-space format, which will be used later on for the simulation:

(31) $$\begin{array}{c} \dot{x}=A\left(q,\dot{q},m,l\right)x+B\left(q,m,l\right)u-{\left[\begin{array}{cc} 0_{1\times n} & G(q,m,l) \end{array}\right]}^{T}\\ x=Cx+Du\\ x={\left[\begin{array}{cccccccccc} q_{0} & q_{1} & . & . & q_{n} & \dot{q_{0}} & \dot{q_{1}} & . & . & \dot{q_{n}} \end{array}\right]}^{T}\\ A\left(q,\dot{q},m,l\right)=\left[\begin{array}{cc} 0_{n\times n} & I_{n}\\ -D^{-1}\left(q,m,l\right)K & -D^{-1}\left(q,m,l\right)\left(C\left(q,\dot{q},m,l\right)+B\right) \end{array}\right]\\ B\left(q,m,l\right)=\left[\begin{array}{c} 0_{n\times 1}\\ D^{-1}\left(q,m,l\right) \end{array}\right]\\ C=I_{n\times n}\\ D=0_{n\times 1} \end{array}$$

#### 2.6.2. Discrete Model

By considering the approximation of the derivative:

(32) $$\dot{x}\approx \frac{x_{k+1}-x_{k}}{T_{s}}$$

we can write:

(33) $$x_{k+1}=x_{k}+T_{s}\dot{x}$$

that is:

(34) $$x_{k+1}=x_{k}+T_{s}\left(A\left(q,\dot{q}\right)x+B\left(q\right)u-{\left[\begin{array}{cc} 0_{1\times n} & G(q) \end{array}\right]}^{T}\right)$$

where $T_{s}$ is the sampling time.

### 2.7. Objective Function

Having the model of the controller and the dynamic model of the multi-segment inverted pendulum on a cart, we could simulate the behavior of our model for any given input set. However, our model was only an analogy to a real structural behavior, so we could not approximate non-zero initial conditions, this being an important criterion. A brief description of the objective function algorithm can be seen in Algorithm 1.
**Algorithm 1:** Objective function.
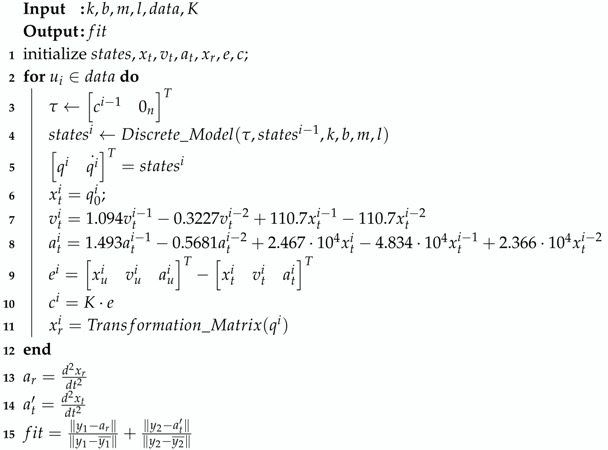


The fit of the objective function was the sum of the normalized mean squared error of the table and roof accelerations between the model and data.

### 2.8. Prediction

One goal of our project was to test our model for prediction and state estimation. Our choice was the extended Kalman filter, because of its performance and ease of implementation.

#### Extended Kalman Filter

The extended Kalman filter is presented in Algorithm 2.
**Algorithm 2:** Extended Kalman filter.
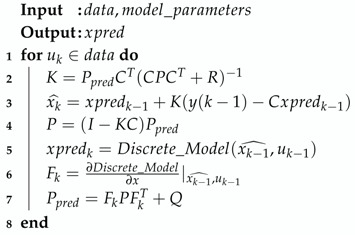


### 2.9. Optimization Stopping Criterion

The stopping criterion used in these optimization algorithms for this project were very simple. Since we have been talking about the model fitness of some data and the objective value of the function was the normalized mean squared error, we could formulate the stopping criterion based on this value. For example, if we wanted a fit greater than or equal to 90%, the NMSE should be less than or equal to 0.1. This stopping criterion was simple and straightforward; however, our algorithm could be caught in an infinite loop if the population converged to a local minimum. That is why another criterion was inserted: the maximum number of generations or iterations. In this project, the stopping criterion consisted of a maximum number of 500 or 1000 generations and a fit of 90%.

### 2.10. Optimization Constraint Handling

The constraints presented in this project were linear constraints of the following form: A·x≤B, where A is nc×dim, nc being the number of constraints and dim the dimension of the problem or the number of parameters to be optimized.

The constraint handling technique was also simple and straightforward and consisted of Algorithm 3.
**Algorithm 3:** Constraint handling.
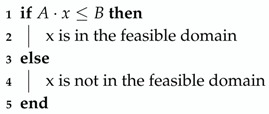


In our case, x was in the feasible domain, which meant that the function would be evaluated in x, and in the case of PSO, it had the chance of being the global attractor, while in DE, it meant that it had the chance of being present in the next generation of candidates. X not being in the feasible domain meant that the function would not be evaluated at that point, so in the case of PSO, it would never have the chance of being the global attractor, while in DE, this meant that it would not survive to the next generation.

### 2.11. Particle Swarm Optimization

The traditional particle swarm optimization algorithm was one of the optimization algorithms used in this project. A brief description of the algorithm is presented in Algorithm 4.

**Figure 8:**
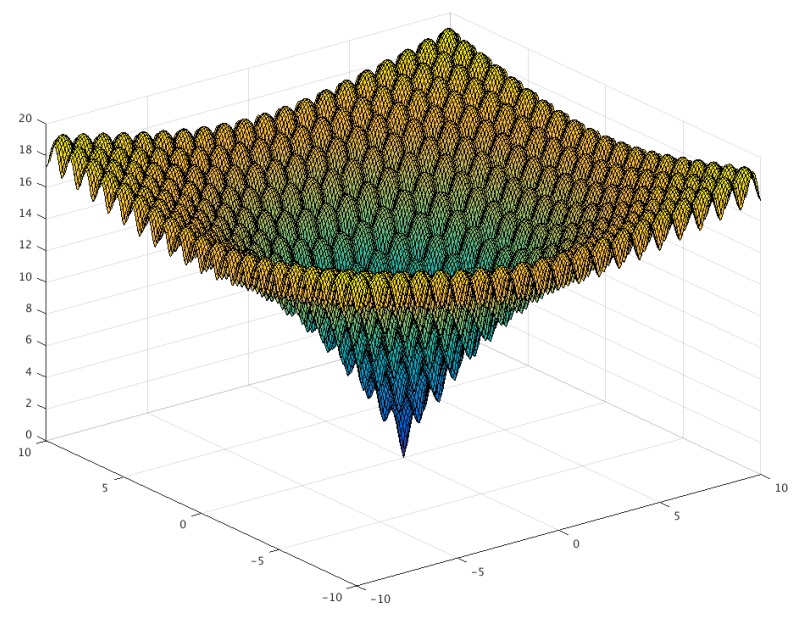
Three-dimensional graph of the Ackley function.

**Figure 9:**
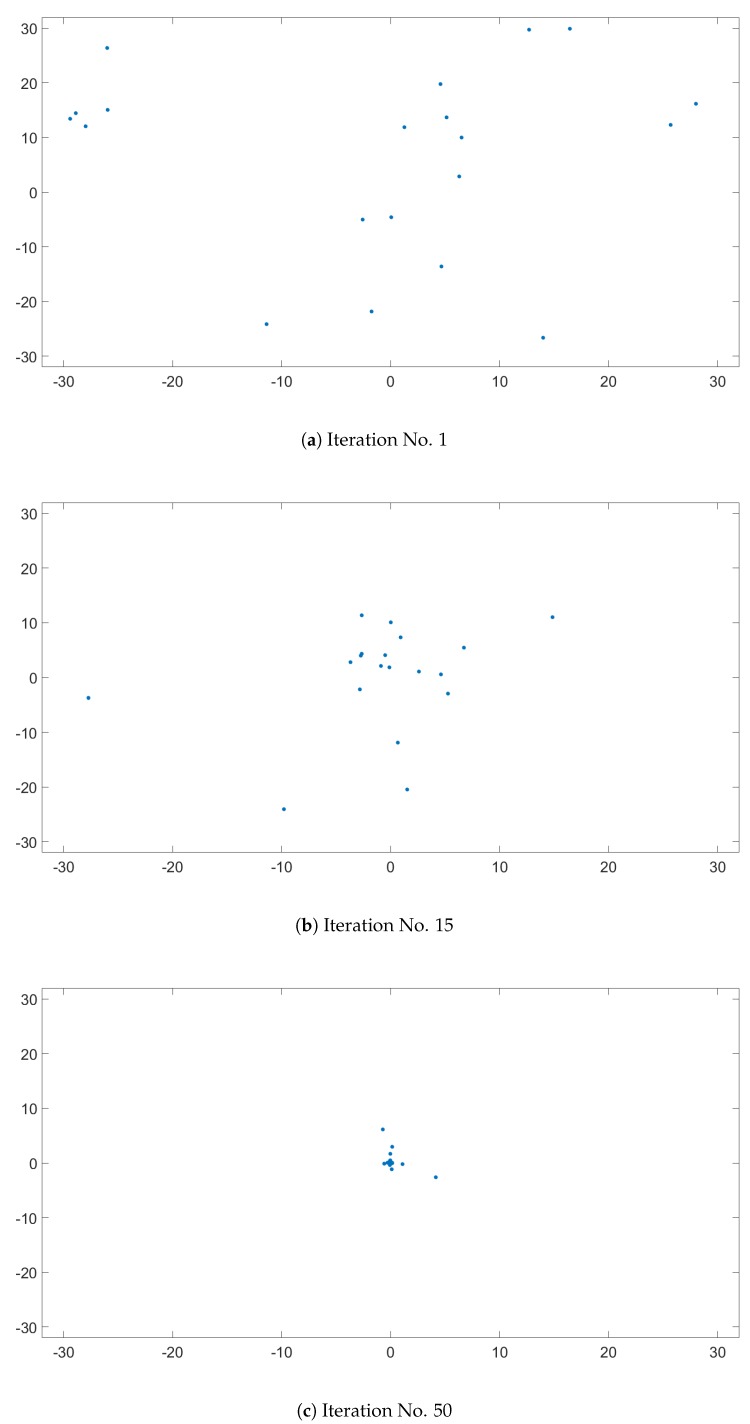
PSO swarm behavior on Ackley’s function.

The Ackley function, seen in Figure 8, is a widely used benchmark function for optimization algorithms, because it has many local minimum points. Figure 9 presents the evolution of candidates of the PSO algorithm on the Ackley function. The algorithm parameters were set to ω=0.749, $c_{1}=1.494$, and $c_{2}=1.494$ with a population size of 20. The candidates were generated in the interval of [−32,32] for each dimension, and no constraints were used. As illustrated in Figure 8, the direction gradient and forward direction were different when the dimension of the Ackley function increased [12]. The global algorithm convergence speed could be detected by this function.

The evolution of the candidates, presented on Figure 9, is illustrated in three subfigures. Figure 9a represents the initial candidate positions, which should be randomly distributed. Knowing that the minimum of Ackley’s function is in [0,0], it is clear in Figure 9b that by the 15th iteration, the candidates were approaching this point. Figure 9c represents the candidates position in the 50th iteration, and it was clear that only a few candidates did not manage to find the minimum.

The variables are the following: *f* is the objective function; lim is a vector of the initial particle limits; A,B are the constraint matrices; dim is the number of dimensions; *n* is the number of particles; $x_{i}$ is a particle; $p_{i}$ is $x_{i}$’s best known position; *g* is the global best position; $c_{1}$ is the cognitive component; c2 is the social component; and ω is the inertia weight.

The inertia weight, social component, and cognitive component were selected from [63], in which different proposals were discussed. This paper discussed four scenarios, which can be seen on Table 4. The population size, according to [76], was not so sensitive to the problems; however a population between 20 and 50 is usually used, except for applications with special needs in this matter.
**Algorithm 4:** Particle swarm optimization.
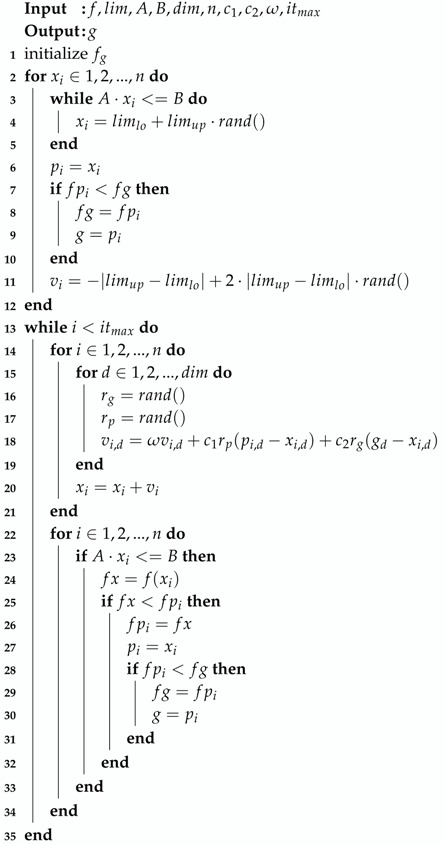


### 2.12. Differential Evolution

As stated in the previous section, in [67], a new global optimization method was proposed called differential evolution. A brief description of the algorithm is presented in Algorithm 5, where *f* is the objective function, lim is a vector containing the initial candidate region, *A* and *B* are the constraint matrices, dim is the dimension of the objective function, *n* is the population size, *F* is the differential weight, Cr is the crossover probability, and $it_{max}$ is the maximum number of iterations. In the algorithm, $x_{i}$ denotes the ith member of the population and $s_{i}$ denotes the ith member of the trial population, whereas *g* is the global best. In this algorithm, the global best did not influence the candidates behavior; it was present only for storing and returning the best possible solution.
**Algorithm 5:** Differential evolution.
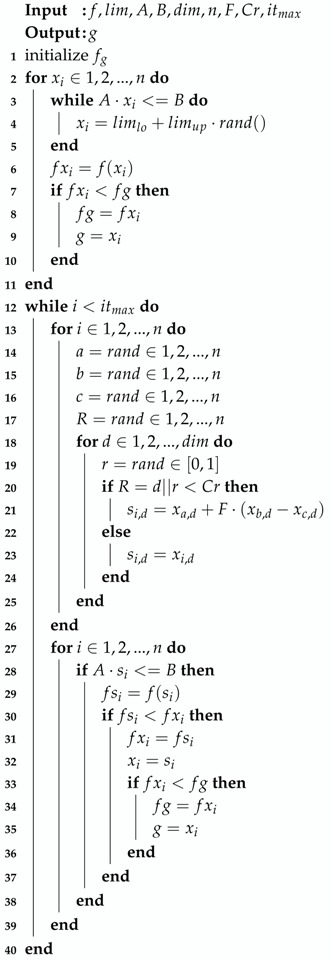


The parameter selection for this algorithm could be performed in many different ways, the best one being a process of meta-optimization. This meant optimizing the algorithm using another optimization algorithm. However, this was not as simple as it seemed, because these algorithms were stochastic; therefore, they would always find the minimum of the function in a different number of iterations. This was why in this project, the selected parameters would be the ones proposed in previous research. As stated in [77], one scenario was choosing a population size between 5·dim and 20·dim with a differential weight *F* of 0.5. Another scenario, according to [78], was to select a population size between 3·dim and 8·dim with a differential weight of 0.6 and the crossover probability bounded between [0.3,0.9]. Furthermore, in [79], it was advised that F∈[0.4,0.95], and as for the crossover probability, it should lie in the range [0,0.2] if the variables were separable and within [0.9,1] when the function variables were dependent. In this project, the above mentioned three scenarios were tested.

## 3. Results

This section discusses the performances of the optimization algorithms, as well as the proposed model using the best parameters obtained after optimization.

The fit is discussed in percentages, using the normalized mean squared error formula.

### 3.1. Optimization Algorithms

#### 3.1.1. Particle Swarm Optimization

**Figure 10:**
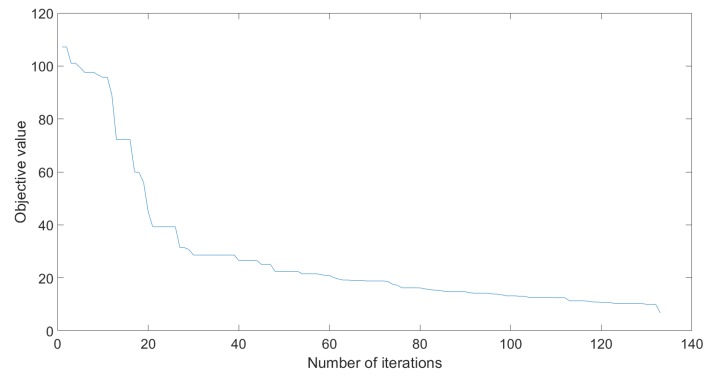
Objective value evolution for PSO.

The tested parameters were the ones proposed in Table 4, for a population of 50, respectively 20. Figure 10 illustrates the value of the objective function over the iterations. The mean number of iterations, standard deviation and success rate can be seen on Table 5 and Table 6.

#### 3.1.2. Differential Evolution

**Figure 11:**
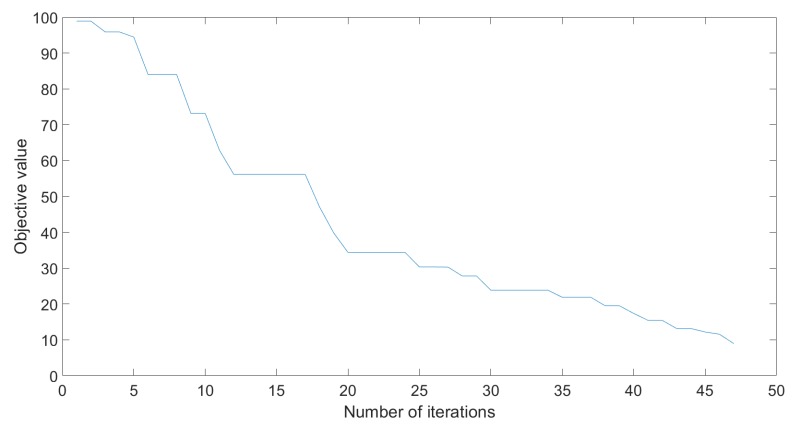
Objective value evolution for DE.

The differential evolution algorithm was tested for a population of 64 (8·dim) 100 times for the parameters seen in Table 7. The evolution of the objective value can be seen in Figure 11.

### 3.2. Proposed Model Fitness for Prediction

**Figure 12:**
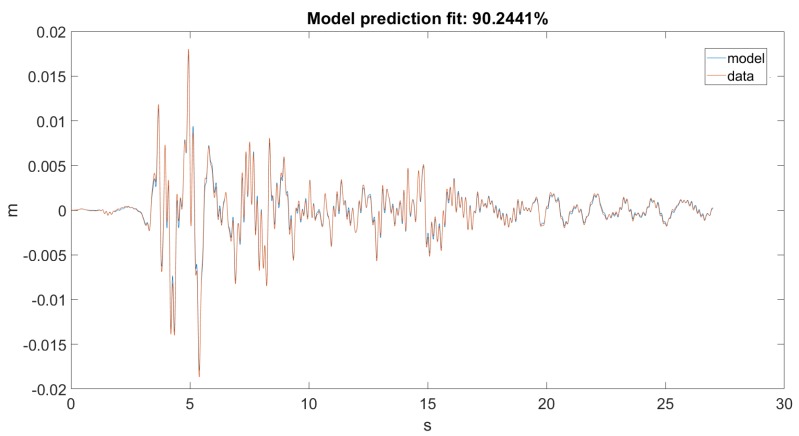
Prediction for Ground Motion 1.

**Figure 13:**
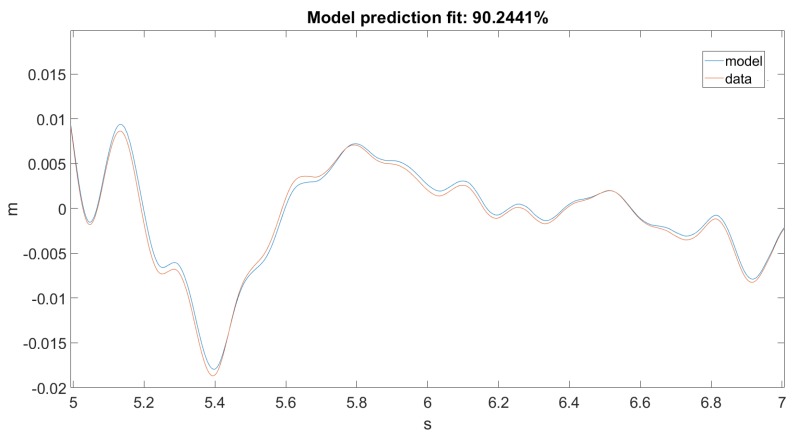
Prediction for Ground Motion 1, zoomed in.

As seen in Figure 12 and Figure 13, the Kalman filter tried to estimate the top displacement of the structure correctly; however, this was not implemented as it should be. As stated, our discrete model returned only angular positions and velocities, but in the dataset, we only had linear accelerations on one axis, which when filtered and integrated, gave us linear positions. The error was calculated between the dataset and the result obtained from the forward kinematic model. However, it would only work correctly if the error was calculated between the dataset and the actual unmodified states of the model.

### 3.3. Proposed Model Fitness for Simulation

**Figure 14:**
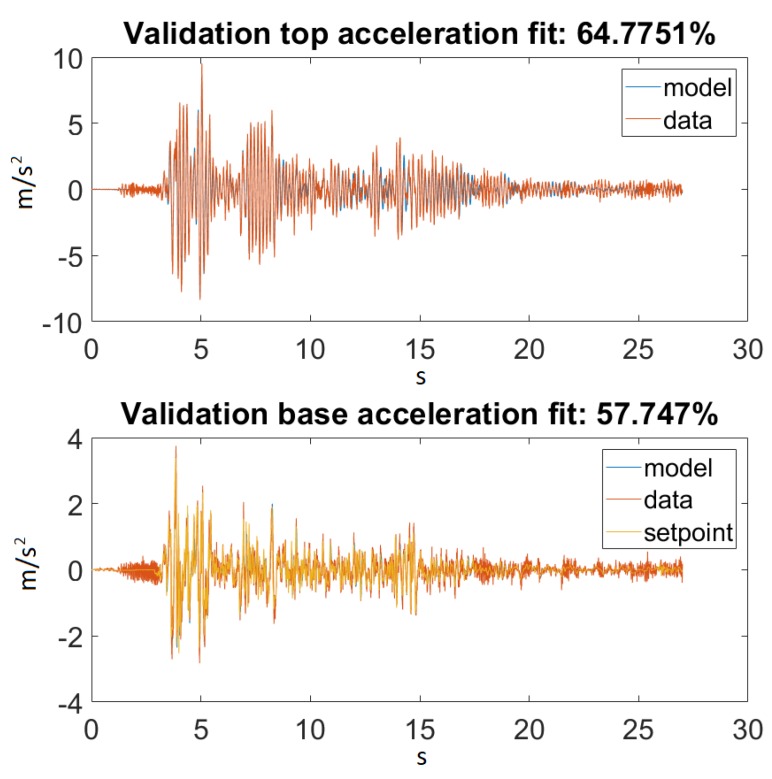
Best fit for Ground Motion 1.

**Figure 15:**
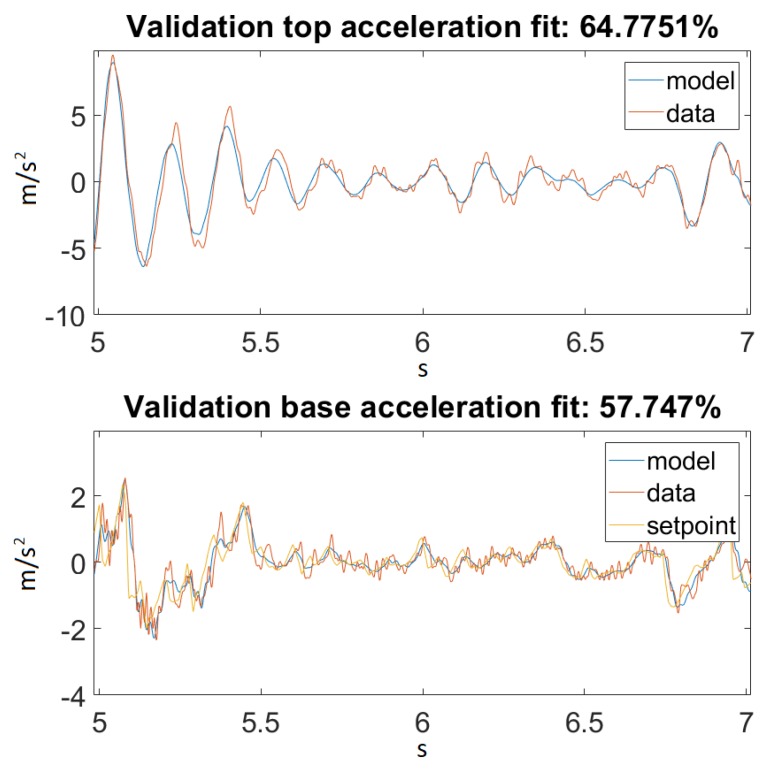
Best fit for Ground Motion 1, zoomed in.

**Figure 16:**
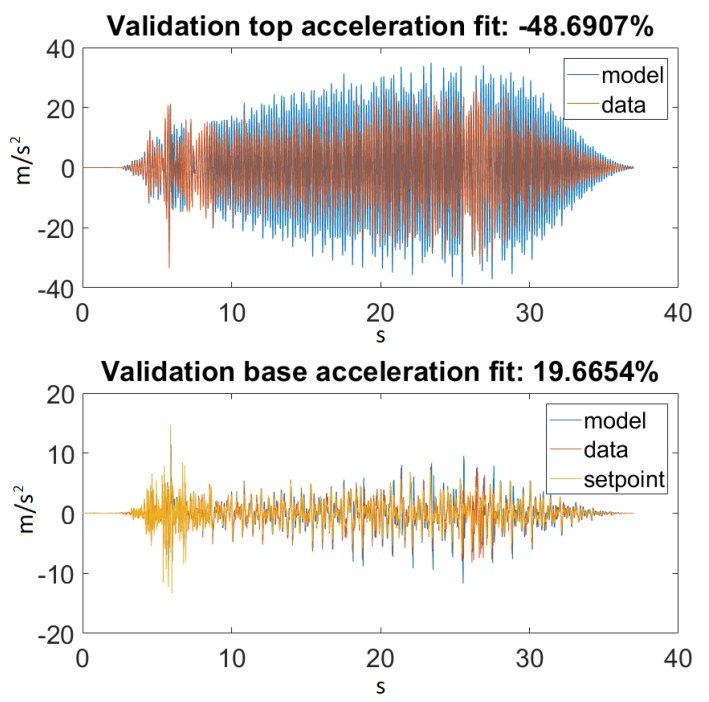
Best fit for Ground Motion 2.

**Figure 17:**
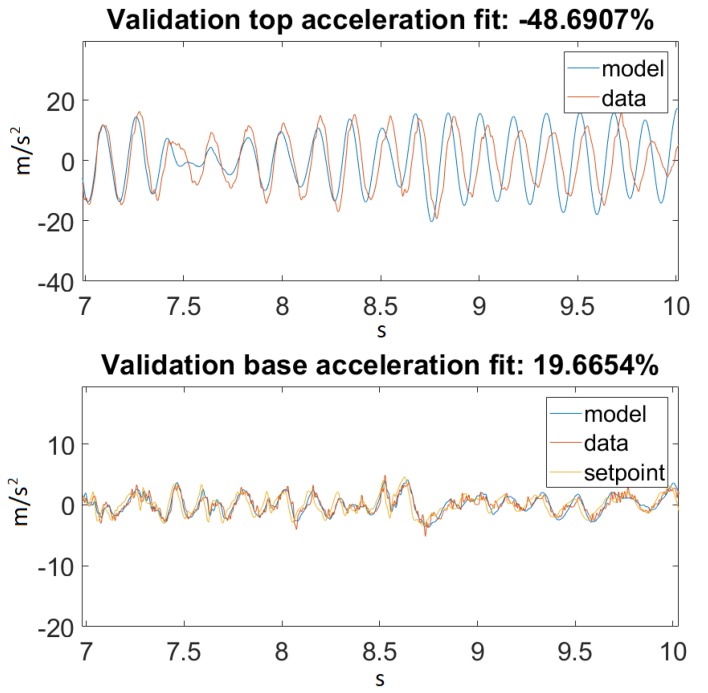
Best fit for Ground Motion 2, zoomed in.

As seen in Figure 14, our model returned an acceptable value for Ground Motion 1. Figure 15 zooms in on the previously mentioned figure for a better visualization. Unfortunately, this could not be said about Ground Motion 2, the reason being that the input was much larger in this case. The performances can be seen on Figure 16 and Figure 17.

## 4. Discussion

### 4.1. Optimization Algorithms

Having discussed the results in the previous section, it was clear that the DE algorithm outperformed its PSO counterpart. The small standard deviation from the mean value is to be noted. Having the figures and tables above, further discussion of these results is not necessary.

### 4.2. Proposed Model

The most important conclusion that can be drawn is that this kind of model, where material nonlinearity was not included, will never have an extraordinary performance for every input range. For smaller inputs, it could nicely approximate the dynamics of a balsa structure, but this also happened because the material behaved linearly for this magnitude of stress.

On the other hand, taking a look at Figure 14 and Figure 16, one can also observe that for Ground Motion 1, it closely followed the structure dynamics, while for Ground Motion 2, it returned an especially poorly fit value. However, looking closely at Ground Motion 2, one can see that it revealed that the model followed the building dynamics, but it was out of phase. A good model would perform approximately the same for every input range. This confirmed that something was missing from our model, which could be the material elastic or damping nonlinearity.

As for the prediction, the extended Kalman filter nicely predicted the displacement of the top of the system; however, this was not entirely correct. Since in our data, we only had accelerations in the shake axis, we could not directly approximate the states. The first reason was that, as stated before, the data contained some low frequency noise, and the accelerations when integrated yielded extremely unreal displacements. This is why the data were filtered with a second order Butterworth high pass filter at 0.8 Hz, as recommended by the manufacturer. The second reason was that, since we only had acceleration on the y-axis and did not have this on the z-axis, we could not compute the equivalent angular positions from those data to calculate the error, since our model’s states were angular positions and velocities. In the algorithm, the error was calculated between the integrated data and the position calculated from the transformation matrix. This was not entirely accurate, because the extended Kalman filter worked properly only if the error was calculated from the states of the system.

## Figures and Tables

**Table 1:** Denavit–Hartenberg parameters.

No.	Symbol	Description
1	$r_{i-1}$	the distance between axes $Z_{i-1}$ and $Z_{i}$ measured on the $X_{i-1}$ axis
2	$\alpha_{i-1}$	the angle between axes $Z_{i-1}$ and $Z_{i}$ measured around $X_{i-1}$
3	$d_{i}$	the distance between axes $X_{i-1}$ and $X_{i}$ measured on the $Z_{i}$ axis
4	$\Theta_{i}$	the angle between axes $X_{i-1}$ and $X_{i}$ measured around the $Z_{i}$ axis

**Table 2:** Shake Table II components.

ID Number	Component	ID Number	Component
1	Stage	9	Sensor circuit board
2	Base plate	10	Right limit sensor
3	DC motor	11	Home position sensor
4	Lead screw	12	Left limit sensor
5	Ball nut	13	Motor leads connector
6	Manual adjustment knob	14	Motor encoder and Hall sensors’ connector
7	Linear guide	15	Accelerometer
8	Linear bearing block	16	Accelerometer connectors

**Table 3:** Denavit-Hartenberg (DH) parameters.

$q_{i}$	d	r	α	θ
$q_{0}$	-	0	$\frac{-\pi }{2}$	$\frac{-\pi }{2}$
$q_{1}$	0	0	$\frac{\pi }{2}$	-
$q_{2}$	0	$l_{1}$	0	-
$q_{n}$	0	$l_{n}$	0	-

**Table 4:** Proposed PSO parameters.

	ω	$c_{1}$	$c_{2}$
Clerc and Kennedy	0.729	1.494	1.494
Trelea	0.6	1.7	1.7
Carlisle and Dozier	0.729	2.041	0.948
Jiang, Luo, and Yang	0.715	1.7	1.7

**Table 5:** PSO performance for *n* = 50.

	Mean Iterations	Standard Deviation	Success Rate
Clerc and Kennedy	116.32	42.24	100%
Trelea	90.13	43.0137	100%
Carlisle and Dozier	110.49	43.394	100%
Jiang, Luo, and Yang	150.2323	63.0657	99%

**Table 6:** PSO performance for *n* = 20.

	Mean Iterations	Standard Deviation	Success Rate
Clerc and Kennedy	184.5	87.33	100%
Trelea	163.9596	88.5733	99%
Carlisle and Dozier	206.1837	98.2121	98%
Jiang, Luo, and Yang	230.8936	91.3517	94%

**Table 7:** DE performance for n = 64.

F	CR	Mean Iterations	Standard Deviation	Success Rate
0.4	0.9	51.1194	7.6228	100%
0.6	0.9	95.2239	12.8261	100%
0.8	0.9	224.4478	27.1624	100%
0.9	0.9	353.1940	42.0434	100%

## Abbreviations

The following abbreviations are used in this manuscript:

PSO	Particle Swarm Optimization
APSO	Adaptive Particle Swarm Optimization
QPSO	Quantum Particle Swarm Optimization
Q-PSO	Quantum behaved Particle Swarm Optimization
PSO-QI	Quantum Infused Particle Swarm Optimization
DE	Differential Evolution
EDE	Elitist Differential Evolution
P	Proportional
PD	Proportional derivative
FF	Feed forward
GA	Genetic algorithm
NMSE	Normalized mean squared error
